# AI-based prediction of protein–ligand binding affinity and discovery of potential natural product inhibitors against ERK2

**DOI:** 10.1186/s13065-024-01219-x

**Published:** 2024-06-03

**Authors:** Ruoqi Yang, Lili Zhang, Fanyou Bu, Fuqiang Sun, Bin Cheng

**Affiliations:** 1 Affiliated Hospital of Shandong University of Traditional Chinese Medicine, Jinan, 250011 China; 2 Shandong University of Traditional Chinese Medicine, Jinan, 250355 China; 3 Jinan Central Hospital Affiliated to Shandong First Medical University, Jinan, 250013 China; 4 Qingdao Municipal Hospital Group, Qingdao, 266000 China

**Keywords:** Protein–ligand binding affinity, Deep learning, Extracellular signal-regulated protein kinase 2, Virtual screening, Natural products

## Abstract

Determination of protein–ligand binding affinity (PLA) is a key technological tool in hit discovery and lead optimization, which is critical to the drug development process. PLA can be determined directly by experimental methods, but it is time-consuming and costly. In recent years, deep learning has been widely applied to PLA prediction, the key of which lies in the comprehensive and accurate representation of proteins and ligands. In this study, we proposed a multi-modal deep learning model based on the early fusion strategy, called DeepLIP, to improve PLA prediction by integrating multi-level information, and further used it for virtual screening of extracellular signal-regulated protein kinase 2 (ERK2), an ideal target for cancer treatment. Experimental results from model evaluation showed that DeepLIP achieved superior performance compared to state-of-the-art methods on the widely used benchmark dataset. In addition, by combining previously developed machine learning models and molecular dynamics simulation, we screened three novel hits from a drug-like natural product library. These compounds not only had favorable physicochemical properties, but also bound stably to the target protein. We believe they have the potential to serve as starting molecules for the development of ERK2 inhibitors.

**Supplementary Information:**

The online version contains supplementary material available at 10.1186/s13065-024-01219-x.

## Introduction

Drug ligands exert specific effects in organisms by interacting with target proteins, and binding affinity is considered the most important criterion for quantifying the degree of interaction between them [1]. Therefore, accurate determination of protein–ligand binding affinity (PLA) is of great significance in the drug discovery process [2, 3]. Traditionally, binding affinities obtained by experimental methods are more reliable, but they are usually expensive and cannot meet the needs of large-scale drug screening [4]. In contrast, computational methods (e.g., molecular docking and molecular dynamics simulation) can quickly prioritize suitable candidates for subsequent experimental testing [5]. However, these physics-based strategies also suffer from low accuracy and high computational overhead [6]. In recent years, benefiting from the rapid development of artificial intelligence technology, some machine learning (ML)/deep learning (DL)-based computational methods have been applied to PLA prediction, among which the performance of ML methods represented by Random Forest and Support Vector Machine relies heavily on the manually extracted features, while DL methods represented by Neural Network are able to automatically capture the feature information from the raw inputs, and their ability to fit complex nonlinear relationships is also stronger [7, 8]. The current DL methods for PLA prediction can be divided into two types: sequence-based methods and structure-based methods.

Generally, sequence information refers to the amino acid sequence of the target protein and the Simplified Molecular Input Line Entry System (SMILES) string of the drug ligand, which can be further extended to the descriptor information (tabular data) of both [9, 10]. DeepDTAF developed by Wang et al. extracts sequence information from three parts (target protein, binding pocket, and drug ligand) to predict PLA [10]. It should be noted that despite the simplicity of the representation, sequence-based methods mostly ignore receptor-ligand interactions. Structural information refers to the three-dimensional structure of the protein–ligand complex, where voxels, graphs, and point clouds are common forms of characterization, and such implicit features are critical for the generation of interactions [6, 11–13]. The structure-aware interactive graph neural network (SIGN) proposed by Li et al. not only preserves the distance and angle information between atoms, but also incorporates long-range interactions into the training process [13]. However, structure-based methods usually lack explicit descriptions (e.g., physicochemical properties). Consequently, researchers have begun to integrate the information learned from multiple modalities. The advantage of fusion models lies in combining complementary representations, thus improving overall performance [14, 15]. Jones et al. constructed a deep fusion model (FAST) inspired by the field of computer vision. This framework improved PLA prediction by combining two features extracted from 3D convolutional neural networks and spatial graph neural networks [15]. However, the computational overhead of 3D convolution is high because it requires invalid voxelization of the 3D structures.

To overcome these limitations, we proposed DeepLIP, a novel DL architecture that employs an early fusion strategy for PLA prediction. Unlike existing methods, DeepLIP represents protein binding pockets and ligands as descriptors, thereby mitigating the redundancy of sequence encoding. Moreover, this architecture departs from the conventional voxelization representation by introducing spatial graphs. This departure not only accelerates computational speed but also allows for a more nuanced understanding of receptor-ligand interactions. By fusing these three disparate levels of heterogeneous information, DeepLIP is able to capture multiple representations and combine them for complementary purposes, ultimately enhancing the reliability of predictions. To the best of our knowledge, the proposed fusion model is the first attempt to integrate such diverse sources of information, underscoring its novelty and potential for advancing PLA prediction. In addition, we explored the integration of our novel approach with other computational strategies for multistep virtual screening to identify promising extracellular signal-regulated protein kinase 2 (ERK2) inhibitors for further investigation. As part of the Ras–Raf–MEK–ERK signaling cascade, aberrant activation of ERK2 has been implicated in many diseases, including cancer, arthritis, and osteoporosis [16, 17]. Therefore, the development of inhibitors against this target may be of great clinical value.

## Materials and methods

### Dataset preparation

For the PLA prediction task, the most commonly used dataset is PDBbind, which consists of three subsets (the general set, the refined set, and the core set) [18]. Typically, DL models achieve better performance when trained on the data-rich general set. However, many studies have shown that training on the refined set improves the model’s prediction on the core set, mainly due to the higher quality of data labeling in the refined set [19]. In this study, in order to fairly compare the performance of these DL models, we referred to the way employed in previous works: the refined set (3,772 samples) and the core set (285 samples) of PDBbind v2016 were used as the training set and the external test set, respectively. More specifically, 20% of the samples from the former were randomly selected as the validation set to optimize the hyperparameters of the pre-training models, and the latter was the benchmark dataset of CASF-2016, a scoring function evaluation platform [20]. Moreover, the non-overlapping refined set (1259 samples) of PDBbind v2020 was used as the internal test set, and the model with the best performance on it was chosen for the benchmark test. It should be noted that the entries within the training dataset (v2016) are not represented in the test dataset (v2020). The statistics of these datasets are summarized in Table 1.

**Table 1:** Statistical summary of the datasets used in this study

Dataset	Purpose	Source	Number
Training set	Training the model	Refined set of PDBbind v2016	3018
Validation set	Optimizing the hyperparameters	Refined set of PDBbind v2016	754
Internal test set	Finding the best model	Refined set of PDBbind v2020	1259
External test set	Benchmark test	Core of PDBbind v2016	285

### Model architecture

**Fig. 1:**
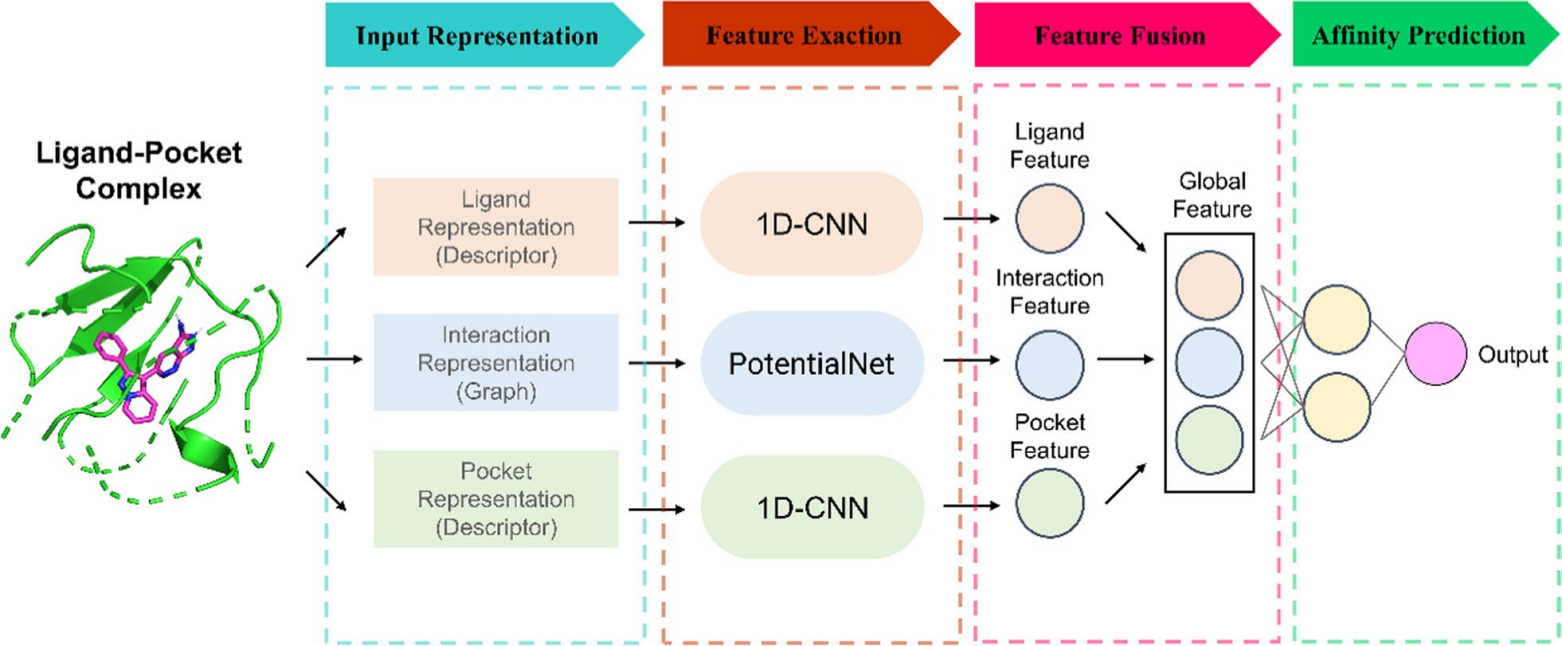
Schematic illustration of DeepLIP, including input representation, feature extraction, feature fusion, and affinity prediction modules

As shown in Fig. 1, the architecture of the proposed DeepLIP comprises four components: input representation module, feature extraction module, feature fusion module, and affinity prediction module. The functions of these modules will be described in detail below.

#### Input representation module

DeepLIP contains three inputs, in which the ligands and the pockets (the cavity inside the protein that directly binds to the ligand, which plays a key role in the strength of PLA) are represented in the form of descriptors. Specifically, we obtained the SMILES string of the ligand (.sdf structure) and the amino acid sequence of the protein binding pocket (.pdb structure) based on the chemoinformatics toolkit Openbabel [21] and the bioinformatics toolkit Biopython [22], respectively, and then calculated the 196 chemical descriptors for ligands and 147 CTD (Composition, Transition and Distribution) descriptors for pockets using the *Descriptors* module of the RDKit toolkit and the *PyProtein* module of the PyBioMed toolkit [23], respectively. In addition, the interactions between the two are represented in the form of graphs, and this process was accomplished using the *PN_graph_construction_and_featurization* module of the DGL-LifeSci toolkit [24]. The details of these representations are listed in Supplementary Table 1.

#### Feature extraction module

The three representations are further extracted by three independent neural network models, in which the ligand features and the pocket features are captured in a similar way. Specifically, it mainly consists of three identical submodules, each containing a 1D convolutional layer, a BatchNormalization layer, and a PReLU layer. Additionally, the interaction features are captured through the PotentialNet architecture proposed by Feinberg et al. [25]. Its highlights are the utilization of distance thresholds as well as gated graph sequence neural networks to learn non-covalent interaction information.

#### Feature fusion module and affinity prediction module

The local features extracted by the three neural network models are concatenated to form a vector based on the early fusion strategy (extracting features from multiple modal information and fusing them), and then the resulting global features are fed into the fully connected layers to predict PLA [26]. It is worth noting that before the fully connected layers, a self-attention layer is introduced to adaptively balance the importance of each representation so that the model can better understand the feature information from different levels. The hyperparameters that need to be tuned in the DeepLIP architecture are provided in Supplementary Table 2.

### Model training

DeepLIP was implemented with the graph framework DGL (version 1.0.1) and the DL framework Pytorch (version 1.13.1), and the hyperparameter search of the model was done using the open-source toolkit Optuna. In addition, since the prediction of PLA is a regression task, we employed SmoothL1Loss as the loss function to train DeepLIP. After training, the model with the minimum error on the validation set was evaluated on the internal test set. The whole process was repeated 10 times with different random seeds, and finally the model with the best performance was used for the benchmark test.

### Evaluation metrics

In this work, three metrics were adopted to evaluate the performance of DeepLIP. Pearson Correlation Coefficient (PCC) is mainly used to measure the degree of linear correlation between the predicted values and the true values, and a larger value indicates a stronger linear correlation between the two. Mean Absolute Error (MAE) and Root Mean Square Error (RMSE) are both indications to assess the gap between the predicted values and the true values; the former represents the overall accuracy of the model, and the latter can estimate the error rate of the model. Normally, the smaller their values are, the closer the predicted values are to the true values.

### Generation of complex conformations

In this study, we used Autodock Vina software [27] to generate docking conformations of protein–ligand complexes and fed them into DeepLIP to re-predict PLA. The molecular docking process involved converting the 3D structures of the target protein (PDB ID: 1TVO) pocket and small molecule ligands to pdbqt format and docking them together. The docking box was positioned near the location of the co-crystallized ligand, and its size was set to 6.25 Å × 6.25 Å × 6.25 Å. In addition, to verify the reliability of the docking process, the co-crystallized ligand was re-docked into the pocket of ERK2. Based on the superposition of the docked conformation and the original conformation (Supplementary Fig. 1), we observed that the spatial orientations of the two conformations were highly compatible, suggesting that the protocol met the requirements. Finally, the optimal binding poses were selected as input for the proposed model.

### Molecular dynamics simulation

The binding stability of five complex conformations with the highest predicted PLA was evaluated using Gromacs software [28], and the procedure was as follows. First, a topology file of the complex was generated based on the CHARMM36 force field [29], and water molecules (SP216) were added to the system along with counterions (Na^+^/Cl^−^). Next, the potential energy of the system was optimized using the steepest descent method, and then the complexes and solvents were sequentially coupled using a canonical ensemble with the temperature maintained at 300 K and an isothermal-isobaric ensemble with the pressure maintained at 1 bar. Finally, a 100 ns molecular dynamics simulation was performed for each system at normal temperature and pressure, while the average binding free energy of the last 10 ns trajectory was calculated by the molecular mechanics Poisson-Boltzmann surface area (MM/PBSA) method [30].

## Results

### Performance of DeepLIP and comparison with state-of-the-art methods

#### Overall performance of DeepLIP on the internal test set

After training the models on the refined set of PDBbind v2016, we directly conducted extensive experiments on the non-overlapping refined set of PDBbind v2020 and selected the best models for the subsequent benchmark test, which is significant in terms of more realistically reflecting the generalization performance and providing a fairer comparison. As shown in Table 2, the optimal performance of DeepLIP on the internal test set achieved 0.698 (PCC), 1.112 (MAE), and 1.372 (RMSE), while the overall robustness of the 10 experiments was desirable. These results illustrate to some extent that DeepLIP has favorable prediction accuracy on unknown data.

**Table 2:** The detailed results of 10 experiments on the internal test set

No.	PCC	MAE	RMSE
0	0.696	1.510	1.907
1	0.724	1.212	1.546
2	0.657	1.197	1.511
3	0.698	1.112	1.372
4	0.592	1.444	1.785
5	0.605	1.451	1.774
6	0.662	1.223	1.617
7	0.697	1.187	1.489
8	0.547	1.478	1.858
9	0.709	1.154	1.457
Average ± standard deviation	0.659 ± 0.056	1.297 ± 0.146	1.632 ± 0.176

#### Comparison of DeepLIP with competitive state-of-the-art methods on the external test set

To more thoroughly evaluate the performance of DeepLIP, we performed a comparative analysis with the highest-performing models in the literature. It should be noted that all models were trained on the refined set of PDBbind v2016 to ensure fairness. As observed from Table 3, our proposed models surpassed the state-of-the-art methods in terms of MAE and RMSE, with the values of both achieving 1.014 and 1.265, respectively. In addition, the value of PCC was also the highest, far exceeding the other models. Interestingly, we found that the models constructed based on fused features generally outperformed the individual models on the external test set. In addition to performance, we also compared the computational efficiency of these models on the core set of PDBbind v2016. As shown in Supplementary Table 3, Pafnucy took the longest time to infer because voxelization required a large amount of computational resources. In contrast, DeepDTAF was the fastest method due to the fact that it used the simplest representation. As the proposed DeepLIP discarded 3D voxels, it achieved a better balance between computational speed and performance and was more competitive compared to other models.

**Table 3:** Performance comparison of DeepLIP with state-of-the-art DL-based models

Model	Architecture	PCC	MAE	RMSE
DeepDTAF [10]	1D-CNN	0.704	1.287	1.629
OnionNet [4]	2D-CNN	0.742	1.120	1.511
Pafnucy [11]	3D-CNN	0.696	1.223	1.617
PointTransformer [13]	PointTransformer	0.753	1.290	1.580
SIGN [12]	GNN	0.797	1.027	1.316
GraphBAR [6]	GNN	0.726	1.241	1.542
FAST [15]	Fusion of 3D-CNN and GNN	0.761	1.231	1.534
DeepLIP	Fusion of 1D-CNN and GNN	0.830	1.014	1.265

All models were evaluated with the External Test Set after training on the refined set of PDBbind v2016 in this study

### Ablation studies of DeepLIP

In order to explore the importance of different input representations for PLA prediction, we conducted a series of ablation studies on the internal test set by removing the pocket features (DeepLI), the ligand features (DeepIP), and the interaction features (DeepLP). As shown in Supplementary Fig. 2 and Supplementary Table 4, removing either the pocket features or the ligand features resulted in a substantial drop in the performance of the model, while removing the interaction features significantly deteriorated the predictive robustness of the model in terms of RMSE and MAE.

**Fig. 2:**
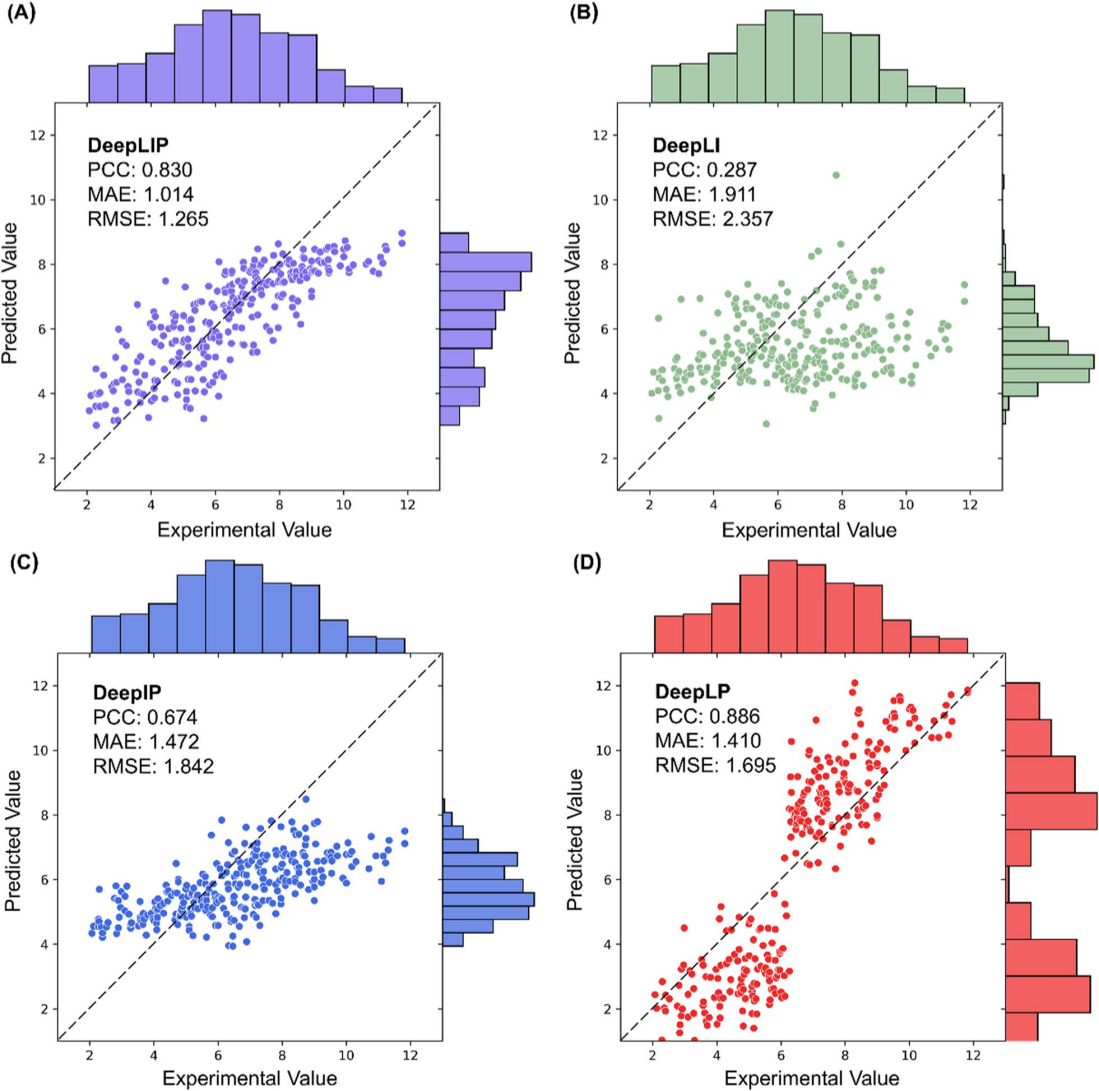
Correlation scatter plot for the benchmark test given by the best models of DeepLIP (**A**), DeepLI (**B**), DeepIP (**C**), and DeepLP (**D**)

Moreover, when we used these models for the benchmark test, their performance also showed different degrees of degradation (Fig. 2). Specifically, DeepLI exhibited the most obvious decrease in performance, with a PCC of only 0.287, as well as high MAE and RMSE of 1.911 and 2.357, implying that the pocket features are critical for PLA prediction. These results are consistent with the findings of Wang et al. [10]. Similarly, DeepIP exhibited a smaller decrease in performance, with a PCC of 0.674, as well as MAE and RMSE of 1.472 and 1.842. We hypothesize the possible reason for this is that PotentialNet retains the partial information about the ligand when extracting the interaction features, and thus the performance degradation due to the lack of ligand features is relatively insignificant. It is worth noting that although sequence-based DeepLP had the strongest predictive correlation on the external test set (the value of PCC was 0.886), its overall prediction gap was still large (the values of MAE and RMSE were 1.410 and 1.695, respectively), which also illustrates the importance of interaction features.

### Visualization of the global features learned by DL models

**Fig. 3:**
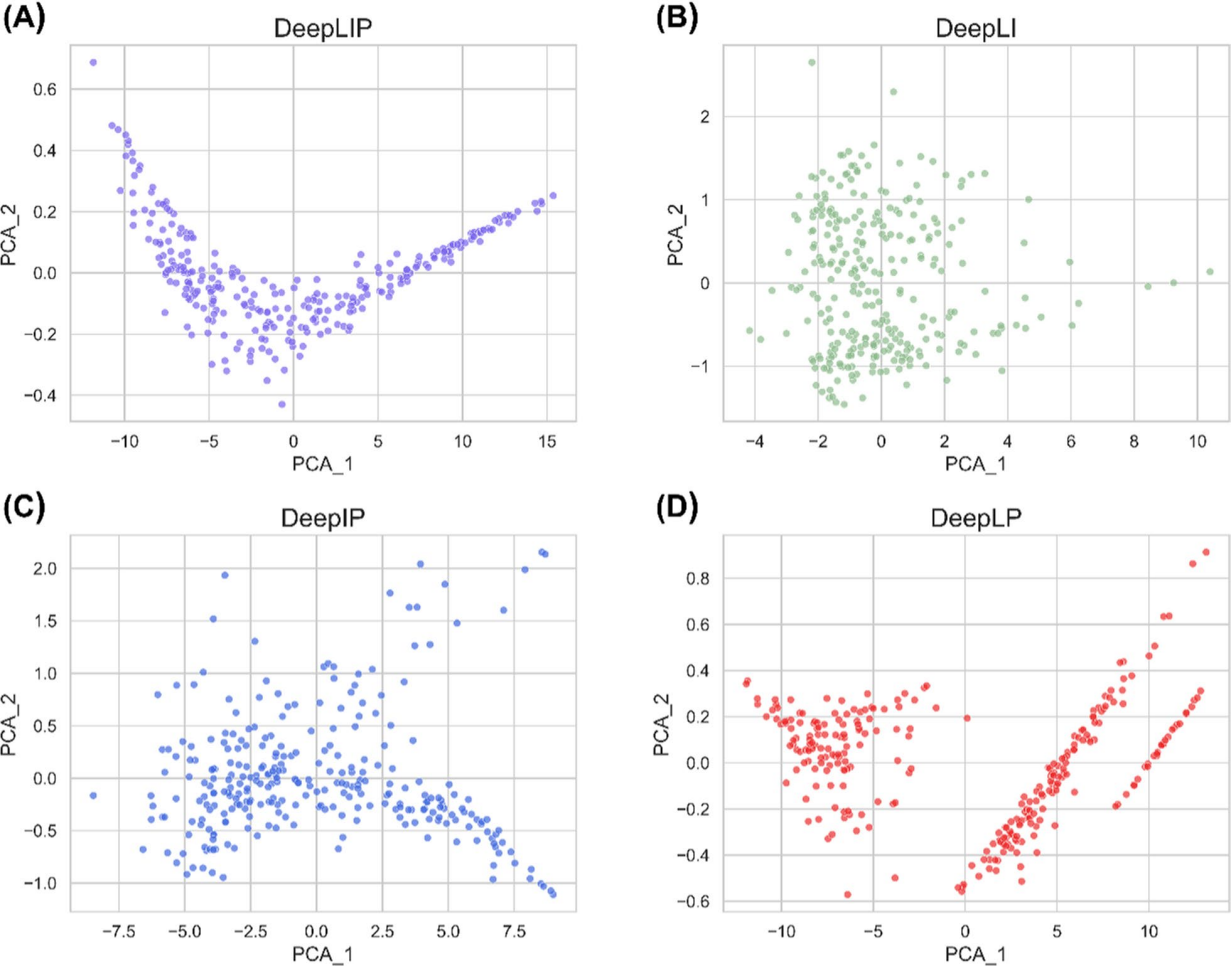
The PCA of the global features extracted by DeepLIP (**A**), DeepLI (**B**), DeepIP (**C**), and DeepLP (**D**) on the external test set

Investigating the black-box mechanism of DL models is a popular research direction in the field of artificial intelligence. In this study, to intuitively understand the information learned by these models, we utilized the Principal Component Analysis (PCA) algorithm to map the captured high-dimensional features into a two-dimensional space. As can be seen in Fig. 3A, the distribution of features extracted after the self-attention layer of DeepLIP was parabolic, which is very easy to fit. In contrast, the distributions of the features captured by DeepLI (Fig. 3B) and DeepIP (Fig. 3C) were more disordered, which well explains their worse performance on the external test set. It should be noted that DeepLP (Fig. 3D) extracted a dispersed feature distribution, which may also be the reason for the strong correlation but low accuracy of its prediction. These results confirm that the fused features learned by DeepLIP can effectively reduce the fitting difficulty and thus improve the overall performance.

### Screening power of DeepLIP and Autodock Vina targeting ERK2

**Fig. 4:**
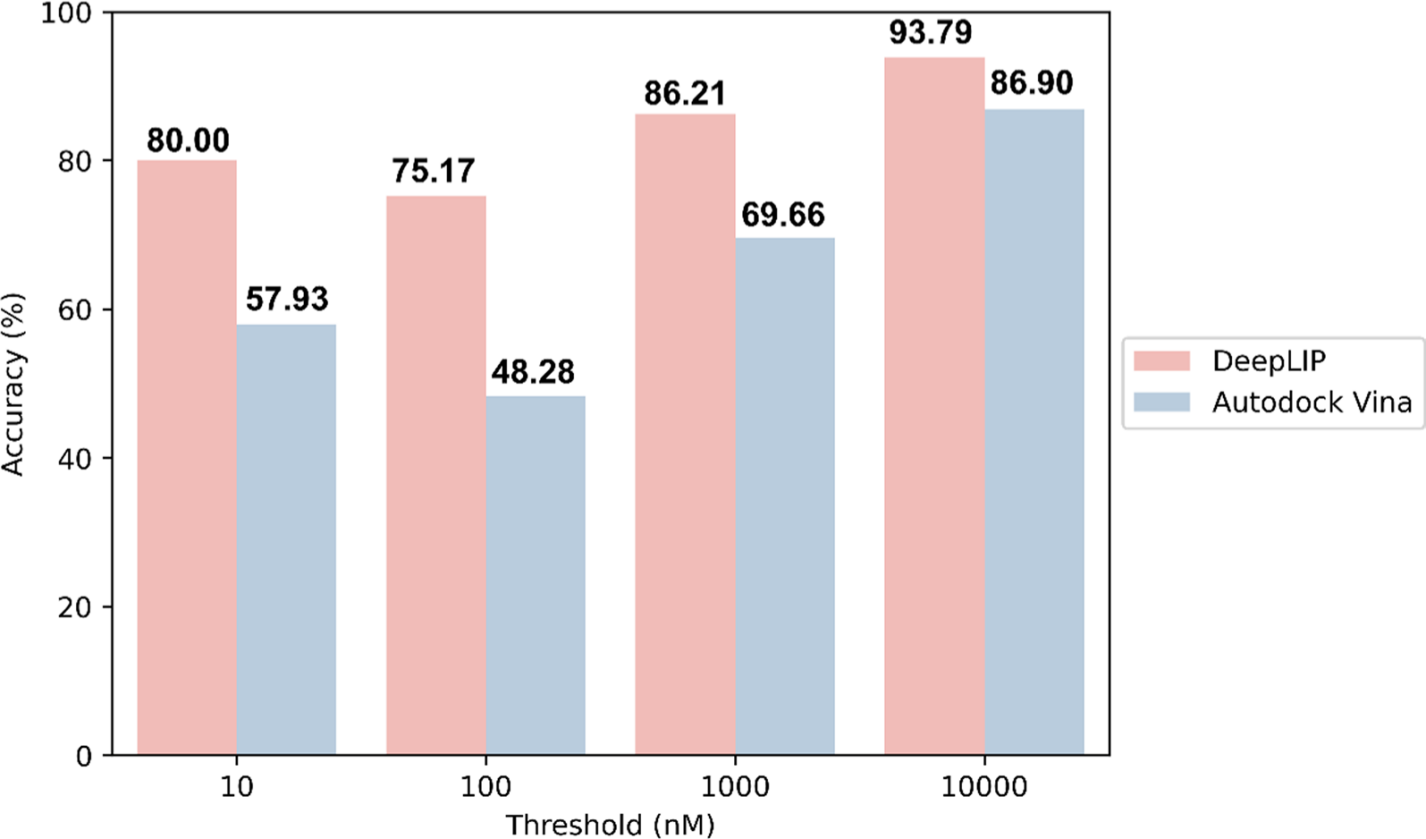
Comparison of the screening performance of DeepLIP and Autodock Vina at different ERK2 activity thresholds

To verify the application potential of the fusion model in real cases, we employed the trained model to perform PLA prediction on 145 ERK2 inhibitors with known activity (the dataset was derived from a previous study by our group), while the prediction results of Autodock Vina were used as a control to simulate the virtual screening process [31]. We assessed the ability of the two methods to discriminate between ERK2 inhibitors/non-inhibitors at different activity thresholds, and their overall accuracy is shown in Fig. 4. Specifically, the prediction performance of DeepLIP at each threshold was significantly better than that of the traditional scoring function, with overall accuracy ranging from 75.17 to 93.79%. In addition, the values of the confusion matrix at different activity thresholds are listed in Supplementary Table 5. We found that the model predicted reliably for ERK2 inhibitors in most cases, but the prediction for ERK2 non-inhibitors was relatively weak. Nevertheless, our proposed DeepLIP can still help researchers discover more reliable inhibitors, thus reducing the cost of subsequent biological experiments.

### Identification of potential ERK2 inhibitors from natural products

**Fig. 5:**
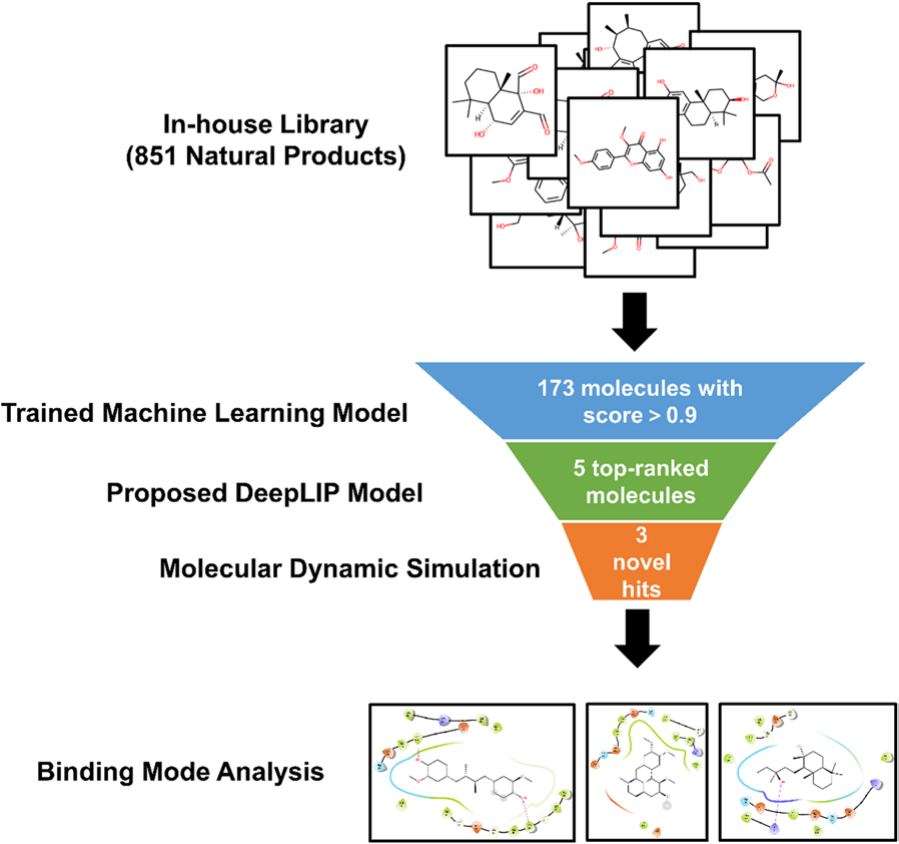
Virtual screening workflow adopted in this study

Given the unique advantages of DeepLIP in the identification of ERK2 inhibitors, we attempted to integrate it with other commonly used virtual screening tools for the discovery of natural product inhibitors against ERK2. In addition, an in-house library containing 851 drug-like natural products manually collected from the relevant literature was used in the overall workflow (Fig. 5). It should be noted that these compounds are clearly named, sourced, and reported with biological activities. We first performed an initial screening based on a previously trained machine learning classification model [31]. Supplementary Fig. 3A shows the distribution of machine learning scores for the 851 original compounds. Subsequently, we selected 173 compounds with scores greater than 0.9 to narrow the screening space, and these compounds were fed into DeepLIP to predict their binding affinity to the target protein. Supplementary Fig. 3B illustrates the distribution of binding affinities for the 173 screened compounds. Finally, five natural products with the highest affinity were selected as potential ERK2 inhibitors. Detailed information about these molecules is summarized in Table 4.

**Table 4:** Details of the five natural products screened from the in-house library

Compound Name	Type	Chemical structure	Source	Reported activity
Schisanhenol	Monophenol		*Schisandra rubriflora*	Anti-apoptosis effect [32]
Dihydroguaiaretic acid	Polyphenol		*Saururus chinensis*	Anti-cancer activity [33]
Rotundine	Alkaloid		*Corydalis tuber*	Analgesic effect [34]
Sclareol	Diterpenoid		*Salvia sclarea*	Anti-tumour and anti-inflammatory activities [35, 36]
Glaucine	Alkaloid		*Glaucium flavum*	Anti-oxidative and anti-viral activities [37]

### Dynamic binding properties of screened hits

**Fig. 6:**
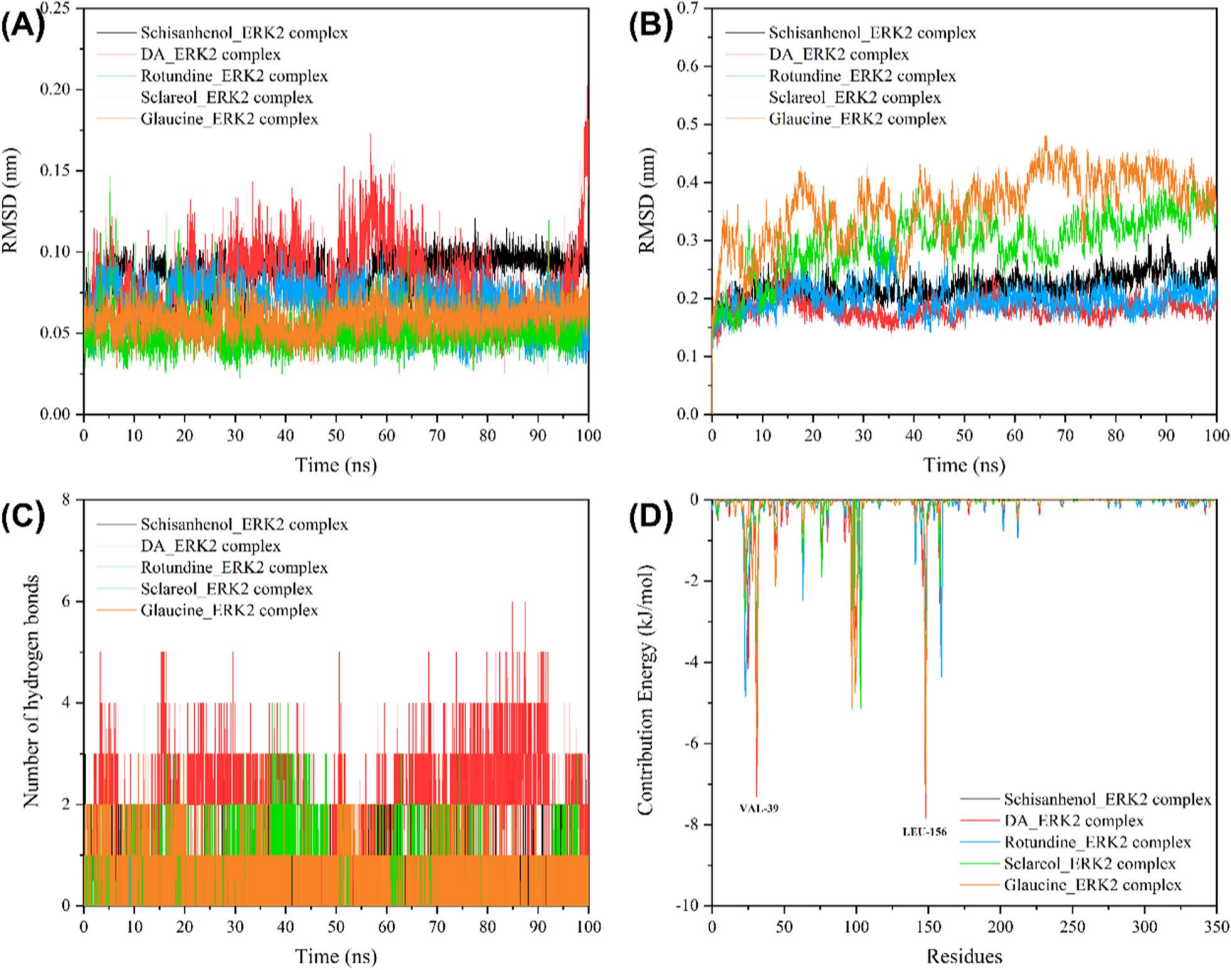
Molecular dynamics simulation of five hit-ERK2 complexes. **A** and **B** show the RMSD values of ligand molecules and protein backbones, respectively. **C** Shows the number of hydrogen bonds, and **D** shows the energy contributions of positive amino acid residues

To further investigate the binding stability between the screened compounds and the target protein, we performed 100 ns molecular dynamics simulations. The Root Mean Square Deviation (RMSD) reflects the movement process of the complex during the simulation, and its drastic fluctuation indicates that the system may be unstable. Figure 6A, B shows the variation of RMSD values with simulation time for ligand molecules and protein backbones, respectively. For the ligand molecule, the RMSD values of Glaucine, Sclareol, Rotundine, and Schisanhenol were stabilized below 0.1 nm, while the fluctuation of Dihydroguaiaretic Acid (DA) was more pronounced due to its longer chemical scaffold and freer movement within the binding pocket. For the target protein, the RMSD values of ERK2 were stabilized at around 0.2 nm when bound to Rotundine, DA, and Schisanhenol, while the fluctuation was greater when bound to Glaucine and Sclareol, suggesting that these complexes were unstable. Therefore, to see if their stability improved or not, the simulation time was extended to 200 ns. As shown in Supplementary Fig. 4, the RMSD trajectory of Glaucine at 100–200 ns was smoother compared to that at 0–100 ns, while the RMSD trajectory of Sclareol still fluctuated drastically. The instability might be due to the fact that it is a strong inhibitor. Overall, these compounds were able to bind tightly to the active pocket of the target protein because the trajectories of both ligand molecules and protein backbones were reasonable. Figure 6C analyzes the number of hydrogen bonds formed between ligand molecules and the target protein during the simulation. Specifically, the highest number of hydrogen bonds were formed between DA and ERK2, while the lowest number of hydrogen bonds were formed between Rotundine and ERK2.

### Calculation of MM/PBSA binding free energy

We also calculated the MM/PBSA binding free energy based on the last 10 ns trajectory. As shown in Table 5, the affinity of DA, Sclareol, and Glaucine for ERK2 was higher than that of the previously identified active compounds [31], and they were promising to be used as potential ERK2 inhibitors for more in-depth studies. Notably, DA and Sclareol have been reported to have anti-cancer/anti-tumor activities, which further confirmed the reliability of the screening results [33, 35]. Subsequently, we decomposed the energy into the interaction between each amino acid residue and the ligand molecule, and extracted the key residues that contributed positively. As can be seen in Fig. 6D, VAL-39 and LEU-156 had a significant effect in most systems, which implies that they may be potential action sites for ERK2 inhibitors. Finally, we compared the binding modes of the three screened inhibitors before and after simulation (Supplementary Fig. 5). DA lost one hydrogen bond with LYS-54 but formed a new hydrogen bond with MET-108 and TYR-36, respectively, the former of which serves as a key residue in the hinge region and is the action site of many reported inhibitors (e.g., GDC-0994 [38] and FR180204 [39]). No hydrogen bond was formed in the Glaucine-ERK2 complex, suggesting that their interactions may be dominated by hydrophobic bonds. A new hydrogen bond was formed between Sclareol and LYS-54 after simulation, and this catalytic residue is located in the gatekeeper region, which is also a common site for inhibitors [40]. In addition, all ligands exhibited different degrees of reduced solvent exposure.

**Table 5:** MM/PBSA binding free energy (kJ/mol) of each ERK2-ligand complex

Ligand	ΔE_vdw_	ΔE_ele_	ΔE_PB_	ΔE_SA_	ΔE_bind_
Schisanhenol	− 123.22 ± 1.12	− 6.64 ± 1.46	94.36 ± 3.12	− 16.74 ± 0.82	− 51.85 ± 2.29
Dihydroguaiaretic Acid	− 103.09 ± 0.84	− 2.83 ± 0.68	53.54 ± 1.73	− 12.53 ± 0.11	− 64.90 ± 1.24
Rotundine	− 149.45 ± 1.41	− 67.01 ± 2.26	177.08 ± 2.91	− 18.63 ± 0.10	− 58.05 ± 1.79
Sclareol	− 142.97 ± 0.99	− 18.92 ± 1.18	99.08 ± 1.62	− 16.81 ± 0.07	− 79.57 ± 1.33
Glaucine	− 158.79 ± 1.11	− 25.80 ± 0.54	109.46 ± 1.16	− 17.75 ± 0.01	− 92.94 ± 1.16

ΔE_vdw_ represent van der Waals energy, ΔE_ele_ represent electrostatic energy, ΔE_PB_ represent polar solvation energy, ΔE_SA_ represent solvent accessible surface area energy, and ΔE_bind_ represent total binding energy

## Discussion

In recent years, an increasing number of computational chemists have utilized DL algorithms to predict PLA in structure-based virtual screening. It has been shown that combining artificial intelligence techniques with classical computer-aided drug design methods facilitates the acceleration of the virtual screening process while reducing false positive rates caused by imbalanced positive and negative samples [41, 42].

In this work, DeepLIP effectively integrates heterogeneous information from three levels. Specifically, it consists of four modules. In the input representation module, ligands, protein binding pockets, and interactions were represented as molecular descriptors, protein descriptors, and spatial graphs, respectively. In the feature extraction module, two sequence features (descriptors) and one structural feature (graph) were extracted by 1D convolutional neural networks and graph neural networks, respectively. In the feature fusion module, the captured local features were concatenated and then assigned appropriate weights by a self-attention layer. In the affinity prediction module, the acquired global features were fed into fully connected neural networks to predict PLA. Experimental results showed that DeepLIP outperformed most existing DL approaches and achieved superior performance on the external test set. Furthermore, the results of the ablation studies showed that each of the local features contributed positively to the prediction of PLA, with the pocket feature contributing the most.

In terms of drug discovery, we performed a first round of virtual screening of an in-house library of our group (containing 851 drug-like natural products) based on a previously trained ERK2 activity classification model. Next, we utilized the proposed DeepLIP model to perform PLA prediction on 173 molecules with high confidence and selected five hits with the highest affinity among them. The results of molecular dynamics simulation showed that three natural products were able to bind stably to ERK2 and had higher MM/PBSA binding free energies than our previously identified active natural products.

However, we cannot ignore that DeepLIP currently has certain limitations. First, the use of binding pockets as local features limits the application of the model in many scenarios, since most of the newly discovered target proteins do not have precise pocket location information. Second, the performance of DeepLIP in large-scale virtual screening needs to be evaluated, and only ERK2 was initially applied in this study. Finally, the prediction results are still a “black box”, and users cannot understand which residues in the interaction contribute more to the predicted binding affinity. In the coming years, we will strive to refine the architecture in the above aspects and optimize the chemical structures of the screened hits as well as evaluate their biological activities.

## Conclusion

In this study, we developed DeepLIP, a novel DL architecture that integrates three types of heterogeneous information: ligands, protein binding pockets, and interactions. Unlike existing prediction models, DeepLIP no longer focused on lengthy sequence coding, but instead represented pockets and ligands in the form of descriptors. At the same time, interactions that are critical for binding affinity were represented in the form of graphs. Extensive experiments on the benchmark test demonstrated the advantages of DeepLIP over other state-of-the-art methods, and the results of ablation studies confirmed the effectiveness of each representation for PLA prediction. In addition, we combined this model with other commonly used virtual screening tools to identify three potential ERK2 inhibitors from 851 drug-like natural products. These compounds had unique chemical scaffolds and remained stable while binding to the target protein. Overall, our proposed DeepLIP provides a valuable tool for the practical application of DL methods in drug discovery.

### Supplementary Information

Supplementary Material 1.

## Data Availability

The datasets generated and/or analysed during the current study are available in the PDBbind repository, http://www.pdbbind.org.cn/download.php. The 3D structure of ERK2 is available in the RCSB PDB repository, https://www.rcsb.org/structure/1TVO.
