# Supplementary material for: AI-based prediction of protein–ligand binding affinity and discovery of potential natural product inhibitors against ERK2
Source: BMC Chem. 2024 Jun 3;18(1):108. doi: 10.1186/s13065-024-01219-x (PMC11145815; doi:10.1186/s13065-024-01219-x)
Supplement: Supplementary file 1 — Supplementary Material 1. [file 13065_2024_1219_MOESM1_ESM.docx]

**Table S1.** The detailed information about the representations used by DeepLIP

| Input | Type | Dimension |
| --- | --- | --- |
| Ligand | Constitutional descriptors  Connectivity descriptors | 106 |
|  |  | 12 |
|  | MOE-type descriptors | 58 |
|  | Molecular property descriptors | 5 |
|  | Topological descriptors | 15 |
| Interaction | Spatial graph | 44 |
| Pocket | Composition descriptors | 21 |
|  | Transition descriptors | 21 |
|  | Distribution descriptors | 105 |

**Table S2.** The hyperparameter information of DeepLIP

| Hyperparameter | Scope | |
| --- | --- | --- |
| The dimension size of the output from PotentialNet in feature extraction module | Stage 1 | [70, 120] |
|  | Stage 2 | [80, 130] |
|  |  |  |
| The number of bond convolution layers of PotentialNet in feature extraction module | Stage 1 | [1, 3] |
|  | Stage 2 | [1, 3] |
|  |  |  |
| The number of kernels for 1D convolution layer in feature extraction module | Layer 1 | [256, 512] |
|  | Layer 2 | [128, 256] |
|  | Layer 3 | [64, 128] |
|  |  |  |
| The number of neurons in fully connected layer in affinity prediction module | Layer 1 | [64, 128] |
|  | Layer 2 | [32, 64] |

**Table S3.** Comparison of the inference time on the core set of PDBbind v2016 (285 samples)

| Model | Architecture | Time |
| --- | --- | --- |
| DeepDTAF | 1D-CNN | about 22 s |
| Pafnucy | 3D-CNN | about 2740 s |
| SIGN | GNN | about 115 s |
| FAST | Fusion of 3D-CNN and GNN | about 79 s |
| DeepLIP | Fusion of 1D-CNN and GNN | about 36 s |

**Table S4.** The impacts of different input representations on the prediction performance of DeepLIP on the internal test set

| Model | Type | PCC | MAE | RMSE |
| --- | --- | --- | --- | --- |
| DeepLI  (removing the pocket features) | Best | 0.355 | 1.502 | 1.909 |
|  | Average | 0.230 ± 0.079 | 1.911 ± 0.182 | 2.382 ± 0.208 |
| DeepIP  (removing the ligand features) | Best | 0.459 | 1.324 | 1.661 |
|  | Average | 0.343 ± 0.101 | 1.815 ± 0.234 | 2.236 ± 0.283 |
| DeepLP  (removing the interaction features) | Best | 0.910 | 1.259 | 1.505 |
|  | Average | 0.668 ± 0.136 | 2.267 ± 0.738 | 2.769 ± 0.898 |

**Table S5.** The values of true positive, true negative, false positive and false negative at different activity thresholds

| Activity Threshold (nM) | True Positive | True Negative | False Positive | False Negative |
| --- | --- | --- | --- | --- |
| 1000 | 120 | 16 | 3 | 6 |
| 1000 | 98 | 27 | 20 | 0 |
| 100 | 75 | 34 | 36 | 0 |
| 10 | 61 | 55 | 29 | 0 |


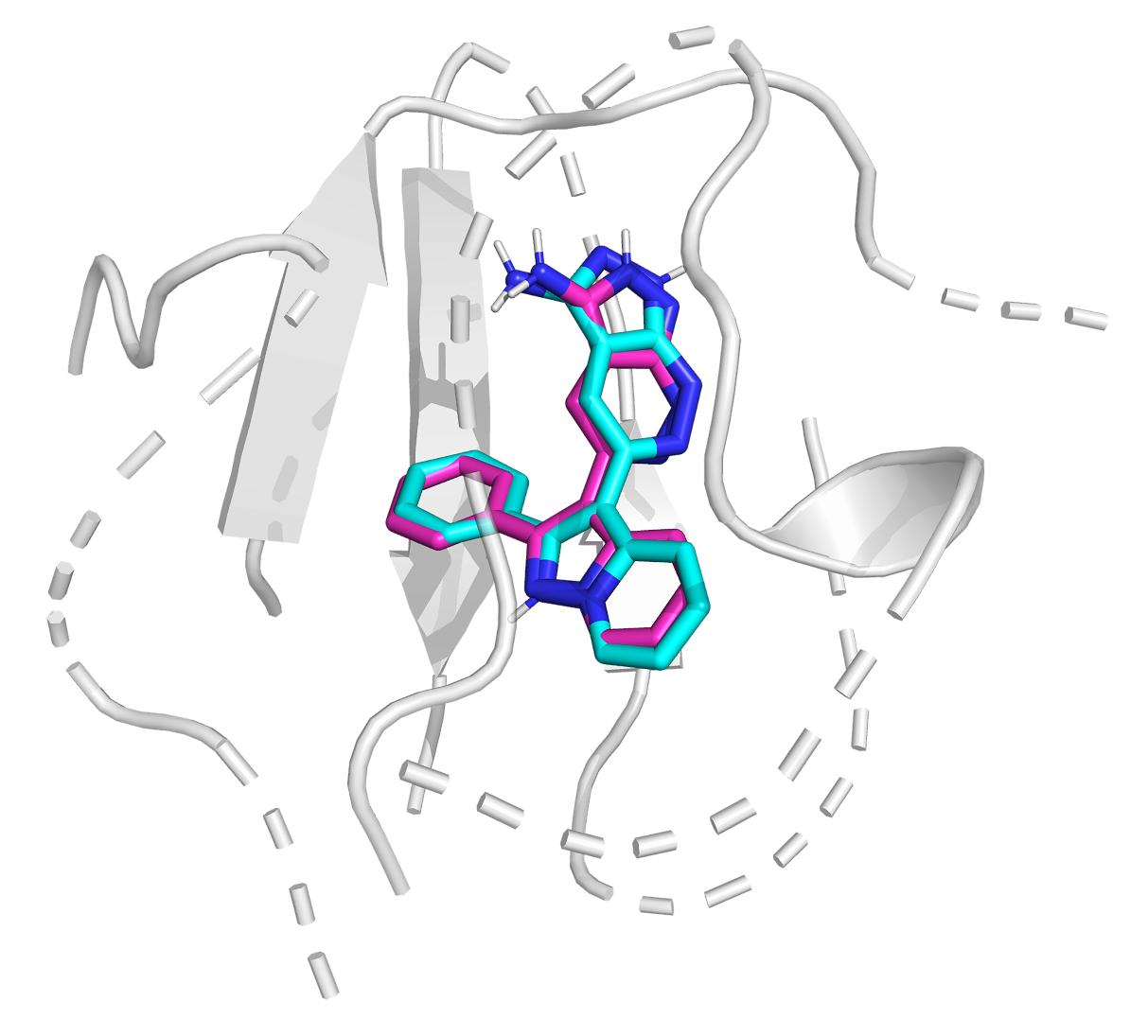


**Figure S1.** Superposition of the docked conformation (pink) and the original conformation (blue) in the pocket of ERK2 (grey). The root mean square deviation (RMSD) between the two conformations was 0.49 Å (less than 2 Å).


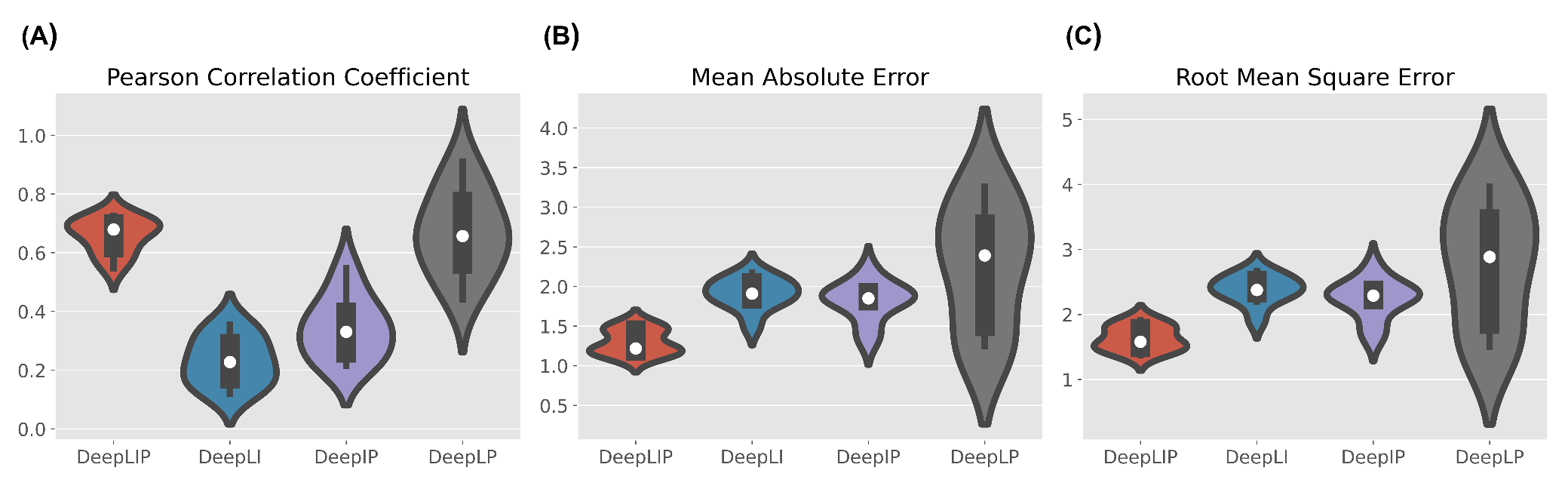


**Figure S2.** Ablation results in terms of Pearson Correlation Coefficient (A), Mean Absolute Error (B) and Root Mean Square Error (C) on the internal test set of DeepLIP, DeepLI (removing the pocket features), DeepIP (removing the ligand features), and DeepLP (removing the interaction features).


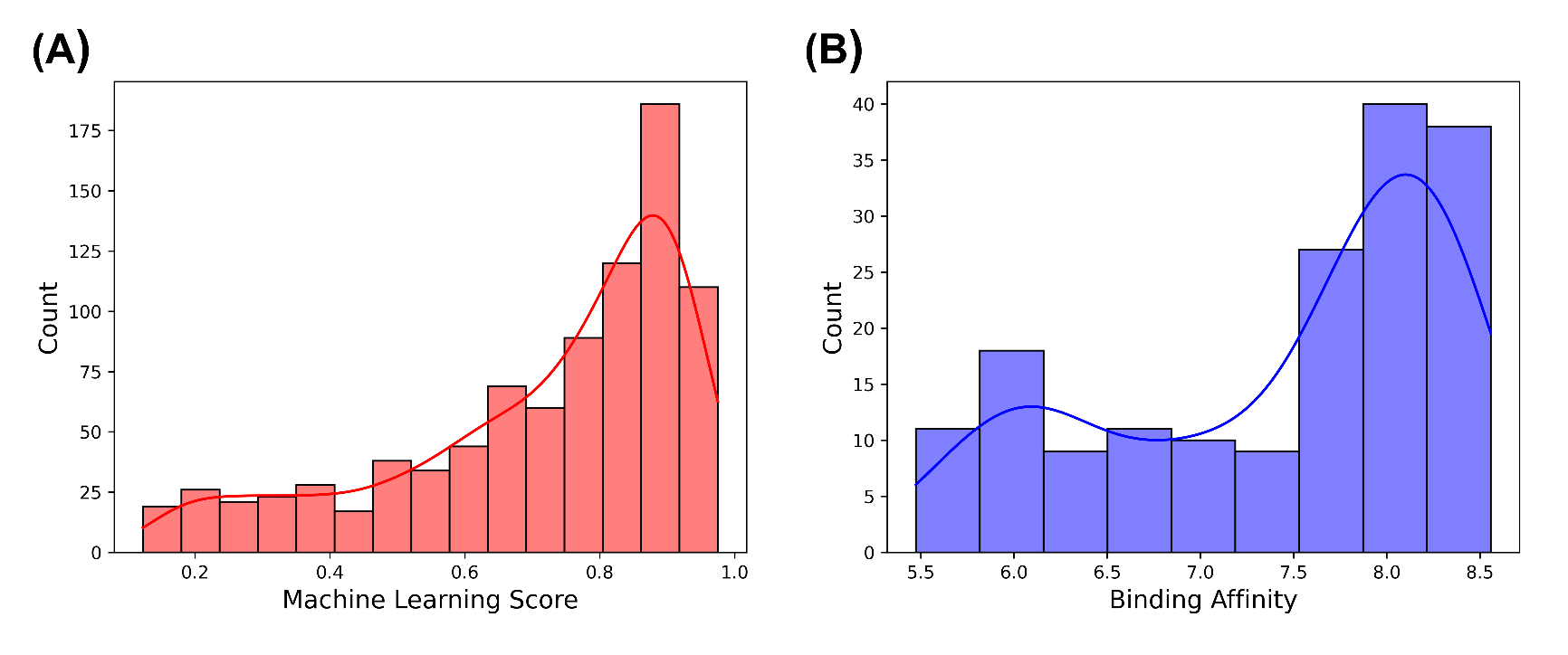


**Figure S3.** Histogram of machine learning score for 851 origin compounds (A) and binding affinity for 173 screened compounds (B).





**Figure S4.** The RMSD trajectories of protein backbones bound to Glaucine and Sclareol.


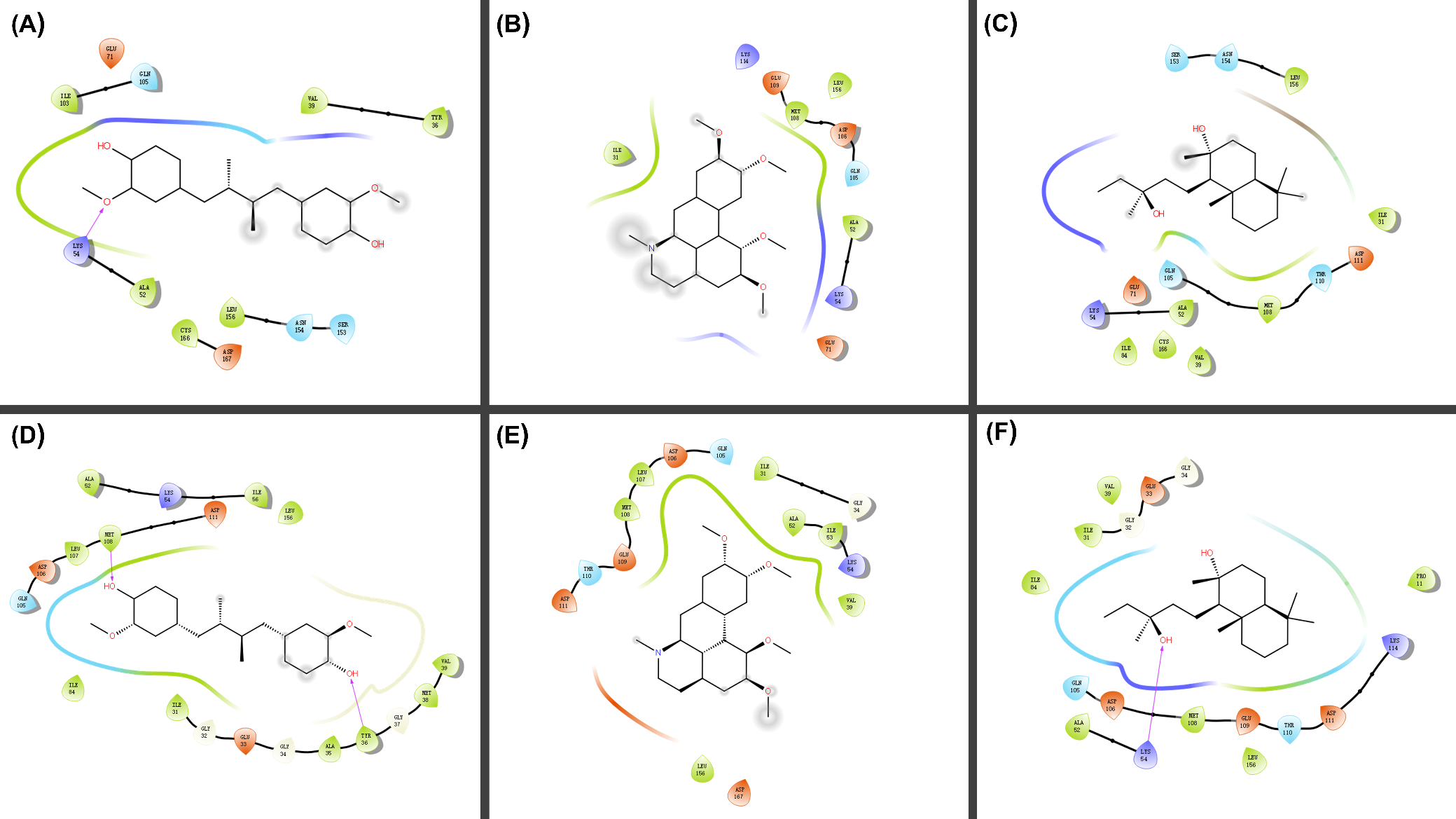


**Figure S5.** Two-dimensional visualization of molecular interactions. (A-C) diagram the binding modes of Dihydroguaiaretic Acid, Glaucine and Sclareol at 0 ns, respectively. (D-F) diagram the binding modes of Dihydroguaiaretic Acid, Glaucine and Sclareol at 100 ns, respectively. Purple arrows indicate hydrogen bonding interaction and bubbles indicate solvent exposure.
